# Teaching higher education staff to understand and support autistic students: evaluation of a novel training program

**DOI:** 10.3389/fpsyt.2023.1264895

**Published:** 2023-12-19

**Authors:** Emma Jenks, Freya Selman, Miriam Harmens, Sarah Boon, Trang Tran, Hannah Hobson, Sarah Eagle, Felicity Sedgewick

**Affiliations:** ^1^School of Education, University of Bristol, Bristol, United Kingdom; ^2^Department of Psychology, University of York, York, United Kingdom; ^3^Health and Social Sciences, University of the West of England, Bristol, United Kingdom

**Keywords:** autism, mental health, higher education, training, intervention, evaluation

## Abstract

**Background:**

Autistic students are particularly vulnerable to stressors within a university environment and are more likely to experience poor mental health than their non-autistic peers. Students’ experiences of stigma from staff and peers, and the masking behaviors they deploy to minimize it, can also result in worsening mental health. Despite these concerns, there is a lack of tailored support for autistic students at university. The current project assesses a co-created training course for university staff focused on debunking stereotypes, educating about the autistic experience at university, mental health presentation among autistic individuals, and practical strategies to improve interactions with autistic students.

**Methods:**

The Autism Stigma and Knowledge Questionnaire [ASK-Q] was administered before and after the training, to examine changes in trainees’ understanding and acceptance of autism and autistic people. Post-training interviews and surveys were also conducted with trainees, covering the impact the training has had on their perceptions of autism, the strategies they found beneficial, and how they will use the materials in future.

**Results:**

There were no statistically significant differences between pre- and post-training scores on the ASK-Q, likely due to ceiling effects as pre-training scores were high. Thematic analysis of interviews identified five themes: value of lived experience; developing nuanced, in-depth knowledge of autism; training as acceptable and feasible; links to professional practice; and systemic barriers.

**Conclusion:**

Although ceiling effects meant there were no changes to participant’s knowledge about autism and autistic people statistically, the qualitative data reveals the extensive benefits they gained from taking part in the training programs. Scoring highly on the ASK-Q did not mean that people could not learn important new information and benefit from the course. This more nuanced understanding of autism led to practical changes in their practice. Listening to and learning from autistic people was seen as particularly important, highlighting the value of co-production. Our results also emphasize the need for varied approaches to evaluating training effectiveness, as reliance on quantitative data alone would have missed the subtler, but impactful, changes our participants experienced. This has important implications for professional practice, both within higher education and more broadly.

## 1 Introduction

Contrary to historical assumptions that autistic people mostly had co-occurring learning difficulties and would not enter higher education (HE), it is estimated that 0.75% of the UK higher education (HE) population had a social or communication difficulty, a category which autism falls under (1). It is worth noting that the total number of autistic students in the UK is not formally tracked by any official body, despite the likely large and growing representation in HE. Increasing research around autism and autistic people in HE settings reflects a recognition among researchers of a need to support this growing population (2), who face additional and unique challenges in these spaces. While transitioning to university can be daunting for most people, autistic students are particularly vulnerable to challenges such as changes to routines, navigating unfamiliar environments, and higher academic or social demands (3).

### 1.1 University experience

Several studies have found that autistic students generally feel comfortable with the academic side of university, compared to other elements of university life (2, 4, 5), which are discussed later in this paper. Some autistic traits, such as attention to detail, strong memory, and different ways of thinking are considered particularly beneficial for university study (3, 4). It is also the case that the ability to focus on a topic more deeply, often one which is a special interest, can enhance students capacity to achieve. This is the case in earlier stages of education (6), and has been mentioned by autistic students themselves (7).

When academic problems do occur, however, this can be a source of emotional distress and anxiety (4, 8). Autistic students may face challenges in the academic setting due to specific autistic traits, such as organization and time management, motivation, and managing course load (2, 5, 9). In Gurbuz et al. (10), students described a difficulty with pacing themselves, sometimes fixating on one subject at the cost of others and the risk of burnout – the potential downside of the ability and desire to ‘hyperfocus’ on a topic or assignment of particular interest. Equally, the perceived pressure to perform in the same ways and to the same standards as neurotypical peers can have a major impact on autistic students, especially if faced with staff who do not understand the extent to which this is a challenge for them (5, 11).

Social communication differences, such as understanding social cues and unspoken social rules, can present a challenge for autistic students at university, as so much of both the formal and informal curriculum depends on these skills and unwritten rules (12). Many report feelings of isolation and loneliness, difficulty in making and keeping friends, social anxiety, and limited or impersonal interactions with their peers (2, 8, 11). Social events at university can often also be inaccessible, with crowded, overwhelming spaces and reliance on alcohol, especially during the first week, known as “Fresher’s Week” in the UK (7, 13). As Fresher’s Week is the time in which most students will get the chance to learn about and join university clubs and societies, many autistic students miss out on those opportunities. Having access to these societies is important, as many autistic students find it easier to interact with people in structured spaces centered around their interests (5, 12).

Outside of social aspects, the physical environment of university can also present difficulties. Loud or bright spaces or busy areas such as lecture theatres and labs can be difficult for students to work in (5, 12, 13). There are, however, solutions that are available in the short term, such as adjusting the lighting (12) and the provision of sensory-friendly spaces (11) – but students have reported difficulty in getting these changes or resources implemented (3). Larger-scale architectural issues, such as narrow, crowded corridors, are possible to address if future building plans are made with neurodiversity in mind.

### 1.2 Stigma and masking

Social communication differences and behaviors can become a source of stigma against autistic people (14). Non-autistic people are less likely to judge autistic people positively or want to engage with them socially (15). Experiences of stigma are commonly reported by autistic people (16–18), with significant negative impact on many areas of their lives.

Reacting to, or fearing, stigma is among the main reasons why autistic people engage in masking, or camouflaging (19, 20). Masking/camouflaging is adopting specific behaviors intended to help an individual fit into a neurotypical environment and hide their autistic traits or social differences (21). It is often not a deliberate choice made by an autistic person, but a response driven by anxiety (22) which can be exhausting and lead to burnout (23).

Masking can have immediate benefits in helping someone to fit in socially and can act as a protective factor against bullying or victimization (16, 24). Some practitioners may encourage masking as a tool for effective socializing (22) but this does not ultimately address the root cause of an unwelcoming environment (20).

The potential harm of suppressing natural behavior in this way has been documented extensively (19, 22, 25). People who frequently mask report losing their sense of self (19, 24) and a sense of disconnection from other people (22). It can also have the unfortunate consequence of the autistic person not being believed when they do reach out for support, because others do not consider them disabled and think that they are able to cope (19, 24). For autistic students, this can mean that it is harder for them to access the supports they are entitled to, or that they face disbelief from staff members when they request reasonable adjustments (7).

Autistic people are more likely to experience mental health conditions, such as depression and anxiety, across the lifespan (26). Anxiety can act as both a trigger and a consequence of masking (22) and extensive masking has been linked to increased rates of autistic burnout, depression, substance use, and suicidality (27–29). In the context of HE, a time associated with increased mental health issues in the general population (30, 31), this relationship may be especially intense as autistic young people attempt to mask their way through multiple new and challenging situations alongside managing independent living and academic pressures.

Indeed, Goddard and Cook (12) found that students were hesitant to disclose to peers who showed little knowledge of autism or relied heavily on stereotypes (such as rudeness, savant abilities, or not feeling emotion). This stereotyping was worsened, in the students’ opinions, by poor media representations of autistic people. If an autistic student seems to be coping well academically, this can be misinterpreted by others to mean that they are not struggling socially (10).

### 1.3 Support

Universities will typically offer traditional academic supports to autistic students, such as extra exam time and separate testing locations. While these can be helpful, they are likely not sufficient for autistic students (8, 32, 33). As discussed above, non-academic concerns also need to be addressed, through options such as peer mentoring, psychological support, and support during the transition to university (5, 11, 32, 34). It is also the case that many autistic students will need individualized rather than generic support options.

Even where adequate supports are available, they are often only provided to students who have a formal autism diagnosis and who choose to disclose it (34, 35). This, however, is not a straightforward decision for students to make and many will wait until they are at a point of crisis before taking that step. They may not consider themselves disabled or in need of support, may be unsure of the process of disclosure, or their circumstances may change during their time at university (3, 4, 10, 35). Students without an official diagnosis may not have access to support at all (11) and those with a diagnosis may experience delays in getting the help they need (4, 9).

As addressing these barriers can be a deciding factor in students’ success and wellbeing at university (3, 11, 33), the current work aims to help staff in becoming more proactive in offering support.

### 1.4 Need for staff training

In addition to the above-outlined aspects of being autistic in HE, many autistic students report negative interactions, or a lack of autism knowledge, among university staff (3, 7, 11, 12). Gelbar et al. (33) describe instances where even staff who studied or taught about autism still did not recognize that autistic students may be in their classrooms. This is not a problem unique to autistic students’ interactions with teaching staff, but also takes place with respect to interactions with those services from whom autistic students might have expected more understanding, e.g., disability services and university mental health services (12, 33).

For students who anticipate, and fear, being treated differently (7, 10), infantilized (12), or considered incompetent (5), an expectation of lack of knowledge and understanding among university staff can prevent students who would otherwise access support from reaching out. Scott and Sedgewick (7) found that, when students were supported by knowledgeable staff with a positive attitude, they felt better supported with their mental health and more comfortable asking for accommodations. Staff with improved knowledge are also more capable of helping their students navigate support systems (4, 11).

Training courses have been shown to make a difference to knowledge and attitudes in university student populations, including around autism (36–38). Jones et al. (39) found that, while their training did not affect implicit bias, such as connecting labels related to autism with negative character traits, it did change explicit bias. This meant that, after the training, participants showed more interest in interacting with, and better first impressions of, autistic people, and were less likely to agree with misconceptions. Similarly, training for university peer mentors can lead to better working relationships between mentors and autistic students. Mentors reported that their new knowledge had been essential to their support role, demonstrating that providing information on autistic students’ needs can be beneficial in helping them access support (38). Therefore, developing training for staff who work with autistic students in the very specific HE context has the potential to significantly positively impact outcomes, both academically and in terms of wellbeing. Participatory training designs, in particular, have been shown to be effective. Gillespie-Lynch et al. (36) ran two versions of a training course and found that, while both showed improvements in stigma, bias and autism knowledge in their sample, the participatory training had a greater impact. Even though the two courses were the same length, participants described the non-participatory version as too long. This suggests that hearing from autistic people, with their own personal stories, was more engaging, potentially resulting in the greater impact it had.

### 1.5 Current study

The current study evaluates a training course designed to address these issues for university staff. This was developed alongside a participatory advisory group (PAG) of autistic students and representatives from the National Autistic Society (NAS) and Spectrum First (an autism training provider). The training was also partly designed and co-delivered by an autistic academic (EJ). Further to this, the PAG suggested the content initially, approved the structure of the training, and recorded interviews sharing their own experiences with the course topics – in line with recommendations for such training to be participatory in nature from studies outlined above. The training was initially designed as a five-week online course, delivered via a virtual learning environment, with a time commitment of approximately 1–2 h per week. The training was partly delivered by an autistic individual and included panel interviews with autistic students, who discussed their own experiences relating to each week’s topic. The order and content of the sessions is outlined below.

*Session One*: introduction to autistic traits, terminology, and theories, emphasizing heterogeneity and the need for individualized support.
*Session Two*: debunking stereotypes, understanding stigma, and autistic masking.
*Session Three*: mental health among autistic people, contributing factors, coping mechanisms, and impact on academic achievement.
*Session Four*: autistic experiences at university and support systems staff could access or signpost to.
*Session Five*: recap of potential challenges for autistic students and how to address these with reasonable adjustments and practical strategies for staff.

To complete the course, participating staff were required to watch a weekly pre-recorded lecture and a video interview with autistic students. Some weeks also had an activity (e.g., a quiz, a visual search task) and optional extra resources, involving YouTube videos, blog posts, journal articles and external websites. Finally, an optional weekly live session was held to allow participating staff to exchange thoughts on the week’s topic, share resources and discuss strategies for engaging with autistic students. This version of the training was run over the summer break, a period in which academic staff tend to have more time available for professional development.

Due to further demand from staff who were not available over the summer, or who could not commit to the five-week course, the training was adapted to a single afternoon session delivered via Microsoft Teams. This version was run over three hours, with the lecture portion delivered live and using shortened clips from the prerecorded student interviews. Further sessions of this version of the training were also delivered in-person at one of the participating universities. Links to the optional extra resources from the longer course were provided after the short course sessions and all staff, regardless of the training version they engaged with, were given a document with key information for future reference.

The current work adopts a mixed-methods approach to address the following research questions:

1. Can training have an effect on autism knowledge and stigma among university staff?
2. Are there advantages to involving the autistic community in developing autism training?
3. How do staff feel that the training has affected their knowledge and practice?

## 2 Methods

### 2.1 Participants

Participants were staff from three different UK universities. The training was initially designed for academic staff with additional roles as personal tutors, who support students throughout the duration of their degree with academic, personal and professional development. As these tutors have frequent one-on-one meetings with their students and act as a first point of contact should any issues arise, the training was tailored toward them. However, enrolment was open to any student-facing member of staff and, due to their interest, the cohort was expanded to include staff in other roles, such as library and disability services (see Table 1). Staff were recruited through advertising sent out in department mailing lists and word of mouth. Through this method of dissemination, it is unclear how many people saw the advertisement, and it is therefore not possible to calculate uptake rates, or the percentage of staff who signed up for the course. More participants took part in the shortened version of the training and full completion rates are listed in Table 2.

**Table 1:** Job roles of each training cohort.

Role type	Full course	Short course	In person
Accommodation	0	1	0
Administration	0	2	10
Careers services	0	1	0
Disability services	2	3	0
Library services	2	5	0
Research	0	0	1
Teaching	5	7	23
Wellbeing services	1	1	0
Other (e.g., technicians)	2	3	4
Other support staff	2	1	2
Total staff	14	24	40

**Table 2:** Course and evaluation completion rates for each course type.

	Full course	Short course	In person	Total
Registered for course	67	49	90	206
Completed training	42	32	52	126
Completed both questionnaires	25	24	40	89

Ethical approval was granted by the University of Bristol School of Education’s Ethics Committee, and all participants were informed of the evaluation study and gave consent before beginning the training.

### 2.2 Materials

Demographic data was collected from all staff before they took part in the training, including age, gender, and role at the university.

All staff completed the *Autism Stigma and Knowledge Questionnaire* [ASK-Q (40)]; twice, pre- and post-training. In the ASK-Q participants select whether they “agree” or “disagree” with a set of 49 statements. Examples include “autism is preventable” or “autism is a developmental disorder.” Their answers are scored as in/correct, for one point per correct answer, and a maximum score of 49.

Once the training was complete, staff from the five-week training were also emailed an invitation to participate in follow-up interviews, the questions for which had been reviewed by the PAG. The interview covered participating staffs’ opinions about the course in general, what they found interesting and motivating, and whether it was easy to navigate. They were also asked how it differed from previous training they may have received, how much time they dedicated to the course, and what they learned. After the short courses, staff were asked to complete feedback surveys. For the first short course, this was a written version of the interview, but for the in-person course, this consisted of two open-ended questions (“which strategies do you think you will use from the course” and “do you have any feedback about the course?”). These changes were made in response to comments from participants about the feasibility of completing the longer feedback survey.

### 2.3 Follow-up data collection procedure

Prior to the interview, staff were sent a copy of the interview schedule and were given the opportunity to ask any questions. Staff had continued access to the course material to review it alongside the questions. Interviews were conducted online using Microsoft Teams with 12 staff members, with both audio and video being recorded with their consent. The system automatically generated a transcript, which was then checked for accuracy. Staff who took part in the interview were paid £20 for their participation. Similar processes were followed for the data from the feedback surveys. These were emailed to staff and were optional for them to complete. Staff were not paid for the surveys as the time commitment was significantly less, and many chose to complete anonymously meaning they could not have been contacted for payment. A total of 54 staff members completed the surveys.

Interview transcripts were analyzed using thematic analysis, as detailed by Braun and Clarke (41). The steps they recommend are as follows: familiarizing yourself with the data; generating initial codes; searching for themes; reviewing themes; and defining and naming themes. Two authors (FeS and EJ) coded all interviews, while all other team members were given a subset of six interviews that were selected at random. All team members initially coded independently of each other and once this had been completed, a consensus meeting was held to decide on the final themes and subthemes to be presented.

## 3 Results

### 3.1 Quantitative results

For the whole dataset (*n =* 89), there were no significant differences between ASK-Q scores before (*M* = 41.16, *SD* = 2.84) and after (*M* = 41.52, *SD* = 2.77) the training, *t*(88) = −1.204, *p* = 0.232.

For the full online version of the course (*n* = 25), there were also no significant differences between pre- (*M* = 41.44, *SD* = 2.42) and post-training (*M* = 42.08, *SD* = 2.33) scores, *t*(24) = −1.104, *p* = 0.281, indicating no change in knowledge or stigma levels among trainees.

There was a significant difference for the online short course (*n* = 24), with pre-training scores (*M* = 41.24, *SD* = 3.82) lower than post-training scores (*M* = 42.42, *SD* = 2.59), *t*(23) = −2.429, *p* < 0.05, indicating that trainees had gained knowledge and reduced stigma.

For the in-person version of the short course (*n* = 40), pre-training scores (*M* = 40.93, *SD* = 2.38) were higher than post-training scores (*M* = 40.62, *SD* = 2.91) but this difference was not significant, *t*(39) = 0.648, *p* = 0.521.

### 3.2 Thematic analysis

**Figure 1:**
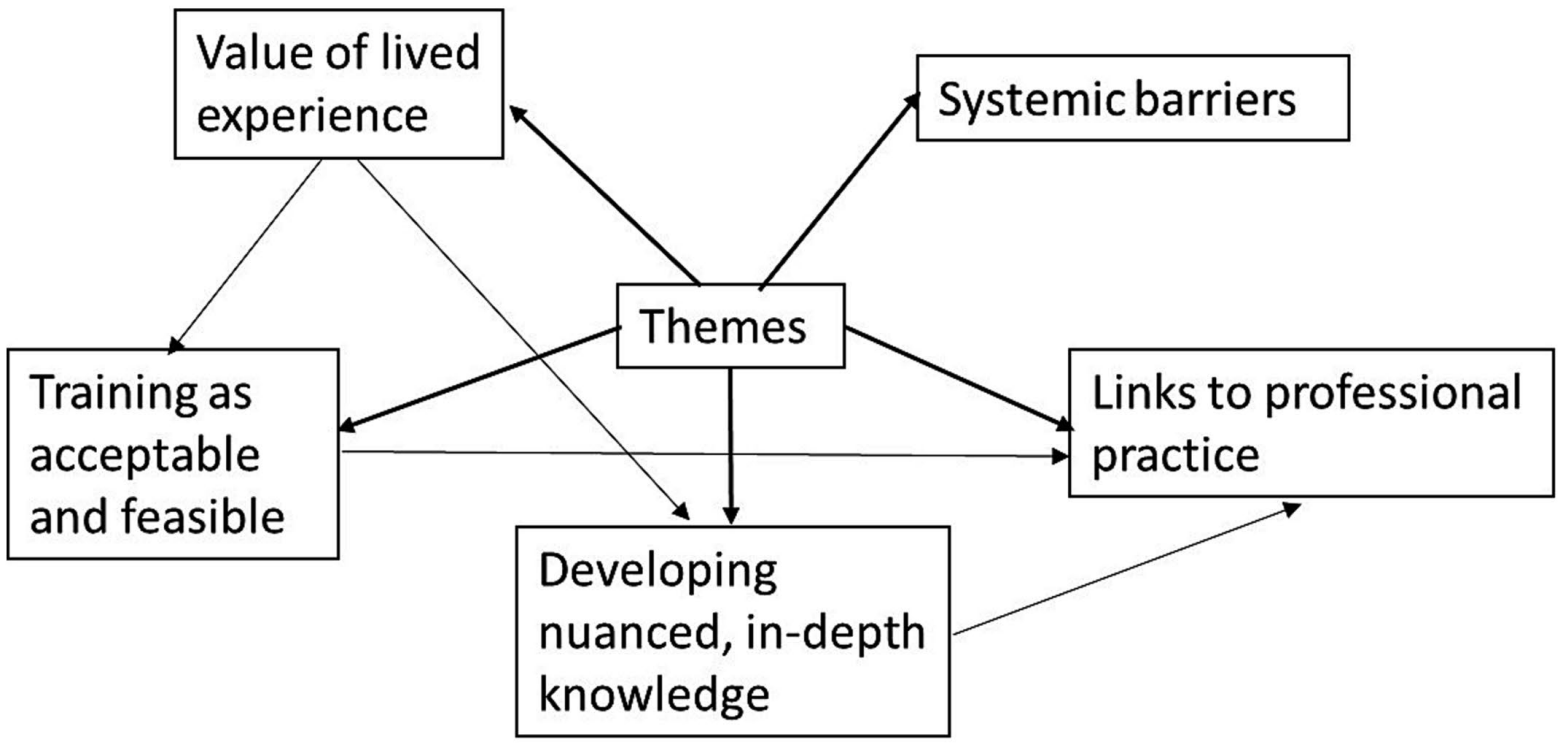
Visual representation of identified themes. Arrows in bold indicate the main themes, thin arrows denote other connections between themes.

Five themes were identified in interviews with participating staff: *value of lived experience*; *training as acceptable and feasible*; *developing nuanced, in-depth knowledge*; *links to professional practice*; and *systematic barriers*. Please see Figure 1 for a visualization of themes and subthemes. Each participant has been given an ID reflecting their course type- “F” for full course (interview participants) and “S” for the short course (survey participants).

#### 3.2.1 Value of lived experience

As mentioned earlier, the training was partly delivered by an autistic individual and included panel interviews with autistic students. Most of the interviewees mentioned this as a key benefit of the training, describing it as “*rare*” (F6), “*powerful*” (F2) and different from previous training they had received. This was echoed in the survey responses, where it was described as “*enlightening*” (S42) and “*a vital part*” of the training (S39). Several mentioned that it was a novel experience to take part in participatory training, and their appreciation for the work this represented from the autistic students and staff member. For example:

*“I recognise there’s a level of… emotional labour that’s needed in that to kind of go ‘here’s my world, here’s my experience, and here are the times where it sometimes is really difficult’” (F2).*

*“was getting to know them and I felt quite a big responsibility to keep watching and keep learning from them, so that helped keep me motivated” (F6).*

The responses from staff who were interviewed also indicated a recognition that it may be more natural for students to cover up their traits when interacting in-person, and that seeing the autistic students and staff member discuss this explicitly was revelatory:

*“People are talking about autism openly in those. That’s almost like that’s what we need to learn because no one does in real life because… that conversation does not really come up. And… the people have just been sort of told to hide it their whole lives anyway so… that’s exactly what we need to see” (F7).*

Seeing the group of autistic people speaking with one another also helped dismiss stereotypes and incorrect expectations from staff. Finding students relatable was a key aspect of this process:

*“they just seemed like normal people I’d wanna be friends with… I expect them to be obviously autistic and because those female ones were just chatting away laughing with you, I was like ‘wait, what? You’re like, you just seem like someone I’d meet at a party’” (F7).*

The inclusion of the phrase “*like normal people*” in the above quote suggests that some staff held ableist attitudes (i.e., that autistic students are not normal) which were being challenged by the inclusion of autistic people in the training. The issue of challenging existing perceptions is discussed further in Section 3.22.

One of the other key perceived benefits of having autistic students involved in the training was that staff were able to put their learning into perspective:

*“sometimes training like this can be very abstract, so adding in those personal experiences and connections to the university was a nice touch” (S3).*

While the lecture material had discussed subjects such as masking and differences in communication styles, the staff who participated in the interviews reflected that these were better illustrated through listening to autistic individuals:

*“They’re telling you about all their struggles but then they seem quite confident the same time” (F7).*

*“How they mask was really interesting and helps me see how different the experiences can be for different autistic people” (S4).*

Finally, staff expressed during the interviews that they had appreciated the personal nature of the student involvement:

*“I did like getting to know the students… you could kind of get a picture of them which was lovely… but also getting sense of how different they were to each other.” (F10).*

#### 3.2.2 Developing nuanced, in-depth knowledge

One of the main themes identified in the responses from both those who participated in the five-week and single-session versions of the course was that the training had allowed them to develop more nuanced and deeper knowledge about autism and autistic HE students. While many had a high level of knowledge going into the training, as shown by the ASK-Q pre-test scores, they still felt that they had learned significant amounts, both in terms of new knowledge and practical strategies they could use.

##### 3.2.2.1 Breaking stereotypes and changing views

While the student interviews helped to present a different image of autistic people than the images that staff on the course were familiar with, the course content itself was also built to debunk stereotypes. Examples brought up by staff during both interviews and surveys included misunderstandings of autistic communication styles and socializing, lack of knowledge of masking and autistic mental health, and thinking that individuals would be *“obviously autistic”* (F7). For example:

*“[I had] slipped into that… Hollywood depiction of autism, you know the white male maths savant. This or the person who’s just supremely non-communicative” (F11).*

*“reinforcing that autism is not bad and does not imply cognitive inability” (S1).*

*“it also promoted a lot of the positive traits that autistic people may have, which was nice because I feel sometimes training can focus more on the barriers and challenges without that balance” (S3).*

There was a range of existing knowledge about autism, and therefore stereotypes, within the group. For some staff, the lecture material was entirely new, although the majority had some prior experience. This meant that some felt that they were carrying no stereotypes:

*“I never thought about these things to begin with even though I think I’m kind of [an] understanding person” (F7).*

While others described realizing that they did hold stereotypes, even if unconsciously or without malice:

*“carrying some unconscious bias” (F3).*

*“preconceived ideas about [autism]” (F4).*

Other staff, for whom the material was familiar, still recognized the benefit of the training in expanding their knowledge and bringing more nuance to the ways they thought about autistic students they encountered:

*“If you went into it thinking well every autistic person’s like Sheldon Cooper aren’t they? It would absolutely change your thinking” (F2).*

*“I would consider myself relatively well-informed about a lot of educational issues, but I was challenged (in a good way) by what was in the course” (S8).*

Some staff had previously taken the need for accommodations personally, or had been hesitant to engage with students whose requests they had previously interpreted as being rude:

*“it’s not a reflection on me when they are wanting more time or asking the questions in those ways it’s a reflection on their needs” (F5).*

*“I’d written them off as this rude person who I did not want to have to engage with and was annoyed if I saw them” (F7).*

Even staff who were knowledgeable about the presence of accommodations had struggled to appreciate their value, and were now beginning to understand the consequences of this:

*“I’ve learned to appreciate it that actually there was some people that do need to have those recordings… being prevented from doing that, or dismissing it, or trying to stop it can actually be more detrimental to the relationship that we have with them” (F5).*

Participating staff noted the conscious effort it took to unlearn these assumptions, both consciously during the training and going forwards in their interactions with autistic (and potentially other neurodivergent or disabled) students:

*“an element of having to sort of reprogram the brain a little bit. And that was definitely what was going on in those couple of weeks” (F4).*

*“it wasn’t something that is really at the forefront of my mind but… it’s definitely something that I’ll think more about and has changed my opinions and my views” (F12).*

##### 3.2.2.2 Building on existing knowledge

Some staff, particularly those working within Disability Services or who were familiar with autistic people in their daily lives, were already knowledgeable about these stereotypes and their inaccuracies. For these individuals, the training provided an opportunity to revise or build on this existing knowledge:

*“[it helped to] clarify certain things or… make it easier for me to explain things to other people” (F2).*

*“reinforced and clarified what I knew” (S1).*

Some staff used the current training to update knowledge that may be outdated or applied to different settings, especially valuing the specific links to the HE context and the ability to compare to training they had on autism previously:

*“nice to see some of the social changes and the language changes and you know to put it in the university context” (F10).*

*“to see how maybe the advice on that manual is no longer valid or is valid still” (F4).*

##### 3.2.2.3 Recognizing students’ individuality

Through the training, staff were able to recognize that any adjustments or strategies learned were not “one-size-fits-all”:

*“It’s not like all neurotypical people are the same and then all neurodivergent people are the same. It just sort of recognising those key differences but there’s a huge variation within that” (F2).*

Throughout the training, staff were able to see the variability in autistic traits, both between people and within the same person in different settings:

*“you could see differences in people, so some of them seemed a lot less talkative and a lot more like someone you would say ‘ohh… they might have autism” (F7).*

*“There are situations in which they… will potentially get themselves anxious, or overly anxious because of the situation they are in, which might not be a particularly anxious situation to somebody else” (F12).*

This highlighted the need to adapt strategies and adjustments to individual students, and the importance of working with autistic students to find what worked for them rather than assuming that the same approaches would work for everyone:

*“there are other ways of working and if this one strategy does not work for them, maybe listen to what they are saying, hear what they are saying” (F1).*

#### 3.2.3 Links to professional practice

Some participating staff intended to continue using the course content and extra materials for reference once the course was complete, to support their ongoing practice and interactions with autistic students in their work:

*“there… to go back over and to…read and to dip in in over a period of time” (F1).*

*“keep for when you come across a case of somebody who wants some support, and actually it’s probably a fantastic resource set to have a look and go ‘oh mental health’” (F9).*

Both the course materials and live session discussions allowed staff to become familiar with accommodations, and different ways of working, that could benefit autistic students. Across all versions of the training, staff noted specific options that they had not been previously aware of, such as a sensory room available at one of the university campuses. Many described a more general practice of simply checking in more often with students and being proactive in their approach.

A common change for staff was in the way they chose to communicate with students, such as providing more time to respond to questions and being more concise. They also discussed implementing their knowledge of conversational scripting and alexithymia into their interactions with students and support plans they pass onto other staff:

*“…being aware not to ask very general things that could… elicit that kind of scripted answer… I’ve definitely mentioned that in a few… support plans that I’ve written and discussions I’ve had with students where they have spoken about sort of not being able to… get in touch really with how they are feeling and difficulty sort of describing it” (F2).*

Sensory and planning needs were also frequently mentioned by staff, such as reflecting more on the space in which they meet students, asking about sensory needs ahead of meetings, and preparing spaces:

*“sometimes people have to go in and look beforehand and… check [a space] out and… walk through it and work out where… everything’s going to be” (F5).*

Contributions made by several staff indicated that accommodations intended for autistic people, particularly in the environment, can be beneficial to the wider student population, for example.

*“In providing a supportive, inclusive environment for the people that we work with and…for the students that access our facilities, actually we are making it more inclusive for everybody” (F5).*

*“If you create a space that is good for neurodiverse people, it’s self-fulfilling in the sense it’s better for everybody. It’s not that you have created a space that is brilliant for a minority but it’s made it useless for everybody else” (F9).*

#### 3.2.4 Systematic barriers

Many staff noted aspects of the structure of their universities that could cause further barriers for autistic students. ‘Crunch points’ around assessment deadlines (*“all of the assessments are due in at the same time,”* F2) and lack of adjustment to assessment requirements were seen as particular issues. The differences between the three institutions in terms of the visibility of neurodiversity, and support available, surprised some trainees – one university had Neurodiversity Champions, and staff there thought this should be standard across HE:

*“I could not believe that in 2022 there was an institution that actually had not engaged with this… I think they were only really starting that engagement because they were thinking about the PR disaster that was facing them” (F4).*

Staff also often discussed the physical environments of university spaces, especially in relation to what they had learned about sensory sensitivities:

*“we have got lots of people going in potentially who are neurodiverse and our facilities… probably were designed… in a decade where we literally did not give a crap about that sort of thing” (F9).*

*“having someone in… the workshop doing mandatory workshop training who cannot take loud noises” (F4).*

Aside from problems directly impacting students, staff discussed systems that prevent them from being able to support students in the way that they would like, such as difficulties in accessing training and barriers to information sharing between relevant departments:

*“in higher education sometimes people have very little time… we did not get great amount of support from our management about you know setting aside time to do it in” (F6).*

*“it was interesting to see what academics were thinking and then disability staff were thinking and how those differed sometimes” (F6).*

*“we can sometimes come up against sort of barriers or just people being difficult where we are trying to recommend adjustments and things and we are basically getting pushback from that… Sometimes it’s perhaps they do not understand why it’s needed and there’s so much in the training that I think would explain why that’s needed. And then sometimes I think it’s a pushback because they do not quite know how to do it or they are worried they do not have time to do it” (F2).*

There were also concerns about being unaware of students’ disabilities, which could be due to university policy around sharing this information:

*“[the training] made me more aware about the very limited flow of information to the lecturers/staff about learning needs” (S44).*

*“I think the students have the assumption that we know and actually we do not” (F11).*

Staff were aware that this issue could also be a result of disclosure decisions or a lack of an official diagnosis, often due to fear of the stigma that has been mentioned in many previous studies about autistic experiences:

*“either [the students] or their families have been incredibly resistant to seeking a diagnosis… because of the stigma presumably and it’s really not been helpful from the students perspective and actually when they do have a diagnosis and we are able to work with them” (F11).*

*“[they] do not want to be that person who has to go and ask for help… you are spending the whole time trying to fit in and not asking for help” (F7).*

There was acknowledgement of the serious consequences of these barriers and a lack of student support, for example on mental health:

*“something needs to be done because like autism does not cause mental health conditions. Being autistic in this world causes mental health conditions” (F2).*

#### 3.2.5 Training as acceptable and feasible

Part of the evaluation of any training is feasibility, as it is important that participants on any course feel able to engage with the materials, complete the training, and that the level is appropriate for the length of the course. Participants on our course generally endorsed the training on these points, although there were more reservations about the short course than the full five-week version.

##### 3.2.5.1 Accessibility

Staff appreciated the structure and delivery of the course, in both formats:

*“both presenters did really well getting across a vast amount of information and context in a comparatively short space of time” (S4).*

*“I knew what I was doing each week. That was really really helpful. And then it meant I knew that I was watching kind of the seminar part and then looking at the student voice and then attending the Q&A session. And then I kind of allocated a bit of time to look at the additional resources as well. So I quite liked that format that really benefited me personally” (F3).*

Staff also discussed how the course content was easy to understand and follow, crucial to the ability to engage with any training and conducive to the learning being retained:

*“it would have been OK to come into it without so much baseline knowledge, because I think it kind of did work it from ground upwards” (F5).*

*“it’s level of pitch was really good” (F8).*

There were a few participants who highlighted that the short course (a single three-hour session rather than the five-week version) felt like it was missing some elements or that the level of content was high for the time allocated:

*“[would like a] longer course to engage in depth with some of the theories underlying the content” (S50).*

*“the length of the initial session… was quite a lot to take in one go” (S8).*

However, this did not appear to impact the accessibility of the course, with a number of staff describing it as clear and informative:

*“it would be hard to cover all important information about autism in 30 h, so for the 3 h provided, I think this was an excellent summary” (S16).*

*“A lot of time, effort, expertise and thorough research and consultation with autistic people (which some autism training really lacks!) has clearly been utilised well to deliver a clear, interesting and informative training program that would be accessible for all staff” (S3).*

*“It’s the best institutional training I’ve experienced” (S4).*

##### 3.2.5.2 Flexibility and time management

The nature of many jobs meant that they were not always able to dedicate a large portion of their time to the course, especially if live discussion in the five-week version clashed with other commitments, which some explicitly lamented:

*“[I] wanted to see what there was in terms of like the breadth of it, and maybe I did not have enough time to go into depth” (F1).*

However, the five-week course structure allowed staff to work at their own pace and fit the resources around their existing schedules, meaning that many still accessed the extra resources and optional readings:

*“I wasn’t sort of stressed trying to fit it in each week” (F7).*

*“I particularly like the fact that it was self-directed time wise, because I could not necessarily do each week… I did two in one week” (F11).*

This flexibility provided more time for reflection for many, enabling them to think about the links between the learning and their professional practice:

*“able to kind of pause it and kind of think about it and sort of make a few notes” (F2).*

*“could go back and do something and then go forwards and then go ohh that connected you know” (F1).*

##### 3.2.5.3 Range of resources

While some staff valued and enjoyed the range of resources provided outside the lectures, others found the amount offered overwhelming – especially those who had less time to peruse them. For some, this became discouraging as they felt that they were not making the most of the training or able to fully engage:

*“we kind of learned about not overstimulating and not over informing. But yet there’s all these resources to look at” (F12).*

*“was just overwhelmed by the quantity which then made it get a little bit lower in the pecking order” (F8).*

*“would feel a little bit guilty if I had not looked at everything” (F3).*

However, other staff emphasized that they *“did not feel pressured to have to go through everything”* (F2), something which was made explicit as part of the training. Additionally, many staff enjoyed the extra resources. The option to engage further, and to follow individual preferences on format (such as choosing to read blogs, or watch videos, or listen to podcasts) was mentioned as a specific strength of the training:

*“it made the training… it sort of brought it to life with those different elements” (F3).*

*“[I] liked the interplay of different resources…something to read, something to watch, something to hear, listen to” (F1).*

*“I personally would not use the social media links… but I thought it was a nice thing to include as many others might find this really helpful” (S3).*

These external materials also provided a direction for further reading when staff had the time to research independently. Many did take up this opportunity, showing that the training became more than a tick box exercise and supported genuine engagement, further learning, and reflection about the topics:

*“I did have one week where I got to look at the extra… YouTube clips and so on and they were very cool … autistic people kind of sharing their experiences.” (F10).*

*“I had the luxury of time. I think I did pretty much all of them and then fell down some Internet wormholes, you know following up on more off of that” (F11).*

*“The presentations, they were really useful to sort of introduce all the concepts but it was more the YouTube videos and the interviews with the students that I thought really engaged me” (F7).*

## 4 Discussion

Autistic students are more vulnerable to stress both during the transition to university and once they are enrolled, and, because of stigma, may feel the need to mask their autistic traits at the cost of their mental health (19, 20, 22, 25). Despite their increased risk of difficulties, they may not be able to access suitable support at university (4, 8, 35). Many encounter staff who hold negative or inaccurate beliefs about autism and are therefore either unapproachable or not able to help (7, 11). The current study evaluates a training course designed to address these issues through improving staff knowledge about autism and thereby potentially make them ‘safe people’ for autistic students to talk to. Staff completed one of two versions of the training, a 10-h course delivered over five weeks (full course), or a 3-h course delivered in one session (short course). This is one of the first papers to report on autism awareness training specifically in the HE context, which is important considering the growing number of autistic students in universities.

Results from the ASK-Q (40) did not show a significant difference in autism knowledge and stigma for the training overall. This could be due to ceiling effects as pre-training scores for all staff were relatively high, with a mean score of 41 of a possible 49. The questionnaire itself was not tailored to the content of the course, so staff may have gained additional knowledge that was not represented by the measure. There was a significant difference for the online short course, which demonstrates that the training could have an impact on knowledge.

For one version of the training, the in-person short course, ASK-Q scores were lower post-training but the reasons for this are unclear. It may be the case that for some of the questions, the training content led staff to mistakenly change their answers. For example, the course covered stereotypes of autism and explained that not all autistic people are boys or men, as is often depicted in media (42). After learning this, trainees could have disagreed with the statement “autism is more frequently diagnosed in males than females,” even though it is correct. Short course participants also had less time between taking the pre- and post-training questionnaires than their full course counterparts, potentially causing a lack of focus or interest in answering the same questions.

While there were no statistical changes in ASK-Q scores pre- and post-training, this does not mean that the training itself was ineffectual. Evaluation interviews and feedback surveys revealed a set of significant benefits and impacts for trainees on both versions of the course.

One of the leading points from the qualitative data was the value of hearing from autistic people themselves, supporting previous work that has suggested benefits of realistic representation and getting to experience participatory training (37, 39). For the current group of trainees, it meant that they were able to place their new knowledge into context. They were able to recognize that students are not usually able to be this open about their concerns and it solidified the idea of the autistic spectrum being much more varied than is generally assumed.

It should be noted that staff demonstrated some ableist beliefs in their post-training interview quotes. While the training did challenge these views, their presence has the potential to impact the approach taken by staff members (43) and whether students feel comfortable discussing their needs with those individuals (7, 11). For instance, one of the participants explained that they did not expect the autistic students to be “normal,” incorrectly framing them as inherently “different” or “other” (44, 45), which could lead to gaps in support within a university context. Autistic people often experience being dismissed because they do not seem to be struggling (24). For students, this can occur because they are masking their autistic traits as well as other challenges such as academic concerns (3). If this continues, particularly for long periods of time (24), it can have a significant impact on mental health (19, 25, 28, 29). Staff therefore need to be more aware that students who appear to be coping may still need support, and this was recognized by those who completed the training.

As noted above, many staff were already familiar with autistic traits and had interacted with autistic students on a regular basis. However, these well-informed staff still felt that they were able to learn from the course. This more nuanced understanding included autistic masking, the recognition of students’ individual presentations and needs, and options for new strategies and supports they could offer. Standard academic supports may not be sufficient for every autistic student (8, 35), and students themselves may not always be aware of supports available or whether they could be of use (4). Their implementation, therefore, relies on staff being proactive in suggesting them and being open to their use. This training improved staff awareness of the supports available, encouraged them to continue learning more and to share the information with colleagues, which could lead to an increase in uptake and therefore improvements in autistic quality of life and academic achievement in HE.

In addition to being aware of university-wide support options, staff also discussed their intentions to adapt their own practice. It has been shown that training that provided an increased understanding of autistic traits helped peer mentors take on approaches that worked for autistic students (39). Adaptations mentioned by staff in the current work included adjusting for social communication and sensory processing differences. The former can prevent students from being able to self-advocate (35), while the latter can make the physical environment of university overwhelming (5, 12). Individual staff making these changes, and potentially spreading them through word-of-mouth to colleagues, therefore has the potential to build better relationships between staff, students, and the university beyond the realm of the training.

The final theme from the qualitative responses was that of systematic barriers affecting autistic students – and staff in supporting them. While some of these – such as sharing good practice and encouraging staff to be more open to student concerns – could be addressed by this training, it needs to occur alongside more large-scale improvements. For example, future university buildings and renovations should be designed in an inclusive manner (12), and policy changes need to be made to improve access to support (46). Training may, however, improve the general inclusivity of campus life and supportive staff have been shown to make a difference to autistic students’ experiences in meaningful ways (7).

Finally, feedback also considered the structure of the training itself. Staff generally found the course accessible and easy to manage, depending on the rest of their workload. There were varying opinions regarding the extra resources provided. While some staff appreciated the chance to dive deeper into the content, others felt overwhelmed and intimidated by the amount, despite these being optional. The time pressures that some staff experienced highlighted the need for university departments to prioritize this type of training and allocate more time for it to their staff. Issues around promoting and ensuring high rates of uptake in this kind of training are discussed in Section 4.2.

### 4.1 Implications for practice

Compared to the general student population, autistic students are less likely to complete their degrees (4) partly due to a lack of knowledge and support from staff (11). The results of this study indicated that, after training, staff felt more confident in recognizing and discussing individual students’ needs. Universities could begin to address the gap in students’ degree completion and improve support systems by introducing further training, and allocating sufficient time for staff to complete it.

Training of this nature can be delivered online, using pre-existing staff training platforms, which would make it simple for institutions to implement. Autistic people should be involved in the development of such training, as they are recognized as experts (47) and their lived experience was described by the current sample as particularly valuable. The course described in this paper is currently being adapted for use at other universities, in addition to charities and other groups responsible for supporting autistic people.

### 4.2 Limitations

This training course was offered on a voluntary basis for staff and required a time commitment either across the summer break or for a single afternoon session without alternative dates. Therefore, it is likely that those who completed the course recognized that they needed to learn more about autism and were motivated to do so. Previous studies (11, 24) suggest that some staff may assume that autistic people may not need support, or that they are unlikely to have autistic students in their classrooms (33). The people who are most in need of such training may not have felt the need or the inclination to attend. This means that the results may look very different, both in terms of the statistical outcomes and the qualitative evaluation, with a different cohort of trainees, something which would be important to test with future studies.

The ASK-Q (40) was not adapted for this training course. Many of the items include questions about children and early interventions that were not relevant for the university-based target group. While it was important to use a recognized and validated measure as an evaluation tool, this may have impacted the results as trainees would not have recognized what they had learned in the questions asked. Those whose scores got worse often changed to incorrect answers on items relating to common autism stereotypes which are based in truth, suggesting that the training did make them question their assumptions, but possibly did not reinforce new knowledge strongly enough. It may be that it is more appropriate for future evaluation studies to use or develop questionnaires which are more closely tailored to the population targeted with the training. It is also important to recognize that the disparity between the number who completed the training, and the number who completed the evaluation surveys, may have impacted the statistical results.

Finally, this study very much focused on attempting to change attitudes and support capacity at the individual, rather than systemic, level. While this is important and has the potential to improve the experiences of autistic HE students with individual members of staff, it does not address the wider systemic issues which have been identified both in previous literature and by the participants in this study. Future work should focus on improving HE systems, from application and transition from school through to assessment and transition into employment or further study. These are the areas with the potential to have larger-scale impact for a wider range of autistic and neurodivergent students overall.

### 4.3 Conclusion

Despite no statistically significant differences between pre- and post-training autism knowledge scores overall, staff still benefitted from the training. The qualitative data showed that even staff who previously had a high level of knowledge or experience working with autistic people were able to learn new information and develop a nuanced understanding of the autistic university experience. This emphasizes the need for multiple methods of evaluation, as a reliance on quantitative data would have missed this more subtle, practical impact.

Participants strongly valued learning from autistic students, which helped put their learning into context and further demonstrated the variance within the autism spectrum. This highlights the importance of both co-production and accurate representation. The training helped to make staff more aware of both existing supports for students and the systematic barriers they face. Trainees discussed plans to alter their approach to working with students, as well as recognizing the importance of sharing good practice. This may allow the course to have a wider, lasting impact through word of mouth and sharing resources with team members. Overall, the training was shown to be feasible and impactful, and shows the importance of HE staff receiving this kind of training to improve how they work with and support autistic students, with potential positive long-term impacts on quality of life and student outcomes.

## Data availability statement

The raw data supporting the conclusions of this article will be made available by the authors, without undue reservation.

## Ethics statement

The studies involving humans were approved by School of Education Ethics Committee, School of Education, University of Bristol. The studies were conducted in accordance with the local legislation and institutional requirements. The participants provided their written informed consent to participate in this study.

## Author contributions

EJ: Data curation, Formal analysis, Investigation, Project administration, Writing – original draft, Writing – review & editing. FrS: Conceptualization, Formal analysis, Writing – review & editing, Methodology. MH: Conceptualization, Formal analysis, Resources, Writing – review & editing, Methodology. SB: Conceptualization, Formal analysis, Resources, Writing – review & editing, Methodology. TT: Conceptualization, Formal analysis, Writing – review & editing, Funding acquisition. HH: Conceptualization, Formal analysis, Writing – review & editing, Funding acquisition. SE: Conceptualization, Formal analysis, Writing – review & editing, Funding acquisition. FeS: Conceptualization, Data curation, Formal analysis, Funding acquisition, Investigation, Methodology, Project administration, Resources, Supervision, Writing – original draft, Writing – review & editing.
